# Right message, right medium, right time: powering counseling to improve maternal, infant, and young child nutrition in South Asia

**DOI:** 10.3389/fnut.2023.1205620

**Published:** 2023-09-04

**Authors:** Arti Bhanot, Vani Sethi, Zivai Murira, Konsan Dinachandra Singh, Sebanti Ghosh, Thomas Forissier

**Affiliations:** ^1^Alive & Thrive, FHI360/FHI Solutions, New Delhi, India; ^2^UNICEF Regional Office for South Asia, Kathmandu, Nepal; ^3^Independent Consultant, New Delhi, India

**Keywords:** counsel, maternal, child, nutrition, diet, breastfeeding, complementary feeding, South Asia

## Abstract

**Introduction:**

Quality counseling can positively impact maternal, infant and young child nutrition (MIYCN) behaviors linked to poor nutrition outcomes. Global guidance includes 93 recommendations on MIYCN counseling.

**Methods:**

A desk review and key informant interviews sought to assess compliance to the recommendations, reach and quality, systemic gaps and opportunities for MIYCN counseling in seven South Asian countries. Ninety-three (93) policies and guidelines, 180 counseling materials and over 50 documents were reviewed; 115 key informant interviews were conducted. Information synthesis captured eight domains. Data from national surveys were analyzed to determine MIYCN counseling reach, quality and association with nutrition behaviors.

**Results:**

Results showed that national guidelines were inconsistent with global recommendations for seven thematic areas. Coverage of contacts points like antenatal and postnatal care (ANC, PNC) with potential to deliver MIYCN counseling was highly variable. Having at least four ANC contacts was significantly associated with consumption of 100+ iron folic acid tablets in all countries. Rates of early initiation of breastfeeding (18% Pakistan to 90% Sri Lanka) were lower than institutional delivery rates, except for Bangladesh and Sri Lanka. PNC contact within 48 h of birth was positively correlated with exclusive breastfeeding in India, Pakistan and Sri Lanka (OR 1.4, 3.1, 3.2). Health worker contacts and wealth status equally influenced child’s dietary diversity in India. MIYCN services were add-on roles for community-based workers, except in India. Supervision mechanisms exist but were not focused on quality of MIYCN services. Counseling resources were predominantly paper based (>70%), had rural-focused messaging on diets and mainly targeted women. Platforms to engage men were largely missing. Health management information systems included indicators on maternal contact points in all countries but not for children. Assessing funding for MIYCN counseling was challenging as costs were subsumed across several budget line-items.

**Discussion:**

The research findings can be used to (1) align country guidance with global recommendations, (2) review workforce responsibilities and capacity building with supervision, (3) assess the need for new counseling materials based on coverage of content, service providers and audience, (4) integrate MIYCN counseling indicators in information systems and (5) include MIYCN counseling services with activities and budget in country plans.

## Introduction

Considerable progress has been made on improving child survival in South Asia. Six of eight countries are either on track to achieve or have already met the Sustainable Development Goal to reduce the under-five child mortality rate to 25 per 1,000 live births or lower by 2030 (1). Nutrition targets, however, will still fall short: Unless nutritional improvements are made, all South Asian countries will miss achieving the World Health Assembly (WHA) 2025 nutrition targets on lowering child wasting to <5% and reducing low birth weight by 30% (2). About 40% of the world’s children affected by stunting and half of those affected by wasting are in South Asia (3). Over 54 million South Asian children are at risk of irreversible physical and cognitive impairment due to chronic undernutrition or stunting, and 27 million are thin or wasted (3). Further, almost 8 million children are dangerously thin or severely wasted (3). The regional prevalence of low birth weight, 27%, is the highest among all regions, indicating poor maternal nutrition (4). Hidden hunger is also the highest among all regions, with an estimated 44–50% of preschool children affected by Vitamin A deficiency (5). Prevalence of anemia continues to be a severe public health problem (≥ 40%) in all South Asian countries among children of 6 to 59 months, except Sri Lanka.

Maternal undernutrition and poor diet and hygiene among young children are critical drivers of child undernutrition in South Asia (6). One in five women is underweight (body mass index <18.5 kg/m^2^) and one in 10 is short statured (height < 145 cm) (7). South Asian countries are off-track to achieve the WHA nutrition target on anemia reduction among all women. Anemia among women remains a moderate to severe public health problem in the region (2, 7). The situation is exacerbated by high rates of adolescent pregnancies in Bangladesh, Nepal, and India (8). While the current prevalence of overweight in children at 2.5% is the lowest in the world, the scenario will worsen due to increased exposure to obesogenic environments and increasing maternal overweight and obesity (2, 9). Prevalence of overweight among women is already exceeding thinness in six of the eight South Asian countries (10). The high prevalence and concurrence of different forms of malnutrition among women fuels an intergenerational cycle of malnutrition. The complex interaction of nutritional deficiencies in the first 1,000 days of life covering pregnancy until a child’s second birthday, and later childhood obesity increases lifetime risk of non-communicable disease (11). Socio-economic disparities persist, with the most vulnerable women and children most deeply affected by undernutrition in both rural and urban settings (5, 12).

Owing to the strong linkages between maternal malnutrition, inadequate diets, and other feeding and hygiene behaviors with child malnutrition, counseling should start early in the first trimester of pregnancy or even before the first 1,000 days (6, 9, 10). Counseling is an interactive process between service providers, pregnant women, mothers, and other family members that can positively influence the practice of recommended behaviors by women and families (13). Governments rely heavily on individual agency to change nutrition behaviors through Social and Behavior Change Communication (SBCC) interventions including counseling (14). In most South Asian countries, women have limited agency with little to no influence in household decision making or access to financial resources. However, regional evidence indicates that even in these settings, quality counseling can positively impact maternal diets and child feeding practices. In India, the likelihood of pregnant women consuming a more diverse diet was 1.5 to 3 times higher among those receiving health and nutrition services and counseling on diets than those not receiving these services (15). Similar findings were noted in Nepal where pregnant women with nutrition knowledge were up to three times more likely to consume a diverse diet (16). More equitable dissemination of nutrition counseling services in the first 1,000 days of life is possible through a strong primary health care (PHC) system. A PHC system aims to bring evidence-based preventive and promotive health care services closer to communities. In the context of the first 1,000 days of life, these include antenatal and postnatal care (ANC, PNC) contacts, care around birth, and all health contacts with infants and young children in the community or first tier public health facilities. However, recent evidence on quality of maternal infant and young child nutrition (MIYCN) counseling during ANC in South Asia revealed that obstacles exist to higher quality counseling. The counseling lasted under five minutes and was more likely to be available at higher tier health facilities than PHC facilities, among other barriers (17).

We identified 93 global recommendations by WHO and UNICEF on counseling for pregnant women, breastfeeding mothers, and children under-two years (18–26). These include 30 recommendations for pregnant women, 24 for breastfeeding mothers and 38 for mother or caregivers of children under-two years. Twelve of the 30 recommendations on pregnancy counseling, six of 25 for counseling breastfeeding mothers and four of 38 pertaining to counseling mothers/caregivers of children under-two are context specific, meaning these recommendations are only applicable when a country meets certain criteria based on population estimates for nutritional status and dietary practices. Other counseling recommendations are described as universally applicable (Tables 1, 2).

**Table 1:** Global recommendations on maternal nutrition counseling.

Recommendation	Counseling focused actions	Context specific	PW	BM
1. Counseling about healthy eating	1.1 Consumption of a variety of locally available foods, including green and orange vegetables, eggs, meat, fish, beans, nuts, whole grains and fruit	No	✓	✓
	1.2 Consume fortified foods (iodized salt and others)		✓	✓
2. Counseling about keeping physically active	2.1 Attain or maintain a healthy weight and/or prevent excessive weight gain	No	✓	✓
	2.2 Take adequate rest		✓	✓
	2.3 Avoid heavy workload		✓	✓
3. In undernourished populations (thinness ≥20% among women), nutrition education on increasing daily energy and protein intake	3.1 Increasing number of meals using locally available foods	Yes	✓	✓
	3.2 Consuming food supplement provided through government program (balanced energy-protein supplement)		✓	✓
4. Daily oral iron and folic acid supplementation with 30 mg to 60 mg elemental iron and 400 μg folic acid	4.1 Importance of daily consumption	No	✓	✓
	4.2 Continued and consistent consumption		✓	✓
	4.3 How to take supplements		✓	✓
	4.4 How to manage side effects		✓	✓
5. Intermittent oral iron and folic acid supplementation with 120 mg of elemental iron and 2,800 μg (2.8 mg) of folic acid once weekly (If daily iron is unacceptable, anemia prevalence among pregnant women <20%)	5.1 Dosage and how to consume	Yes	✓	✓
	5.2 How to manage side effects		✓	✓
6. Antenatal multiple micronutrient supplement in context of rigorous research	6.1 Dosage and how to consume	Yes	✓	
	6.2 Continued and consistent consumption		✓	
7. In populations with low dietary calcium intake, daily calcium supplementation (1.5–2.0 g oral elemental calcium)	7.1 Importance of daily consumption	Yes	✓	
	7.2 Continued and consistent consumption		✓	
	7.3 How to take supplements		✓	
	7.4 How to manage side effects		✓	
8. Among pregnant women with high daily intake of caffeine (>300 mg) reducing caffeine	8.1 Reduced frequency of caffeinated drinks	Yes	✓	✓
	8.2 Avoid tea/coffee with meals		✓	✓
9. Breastfeeding	9.1 Importance of breastfeeding	No	✓	✓
	9.2 Immediate and uninterrupted skin-to-skin contact after birth		✓	✓
	9.3 Early initiation of breastfeeding		✓	✓
	9.4 Correct positioning and attachment		✓	✓
	9.5 Exclusive breastfeeding till completion of 6 months of age		✓	✓
	9.6 Responsive breastfeeding		✓	✓
	9.7 Risks of giving formula, breast milk substitutes, using bottles, teats and pacifiers		✓	✓
10. Counseling on handwashing at critical times and food hygiene	10.1 Handwashing at critical times	No	✓	✓
	10.2 Safe handling, preparation, and storage of food		✓	✓
11. Dietary advice and information on factors associated with constipation for prevention of postpartum constipation	11.1 Consumption of high fiber foods and fluids	No		✓

^1^The International Federation of Gynecology and Obstetrics recommends planned 30 min exercise daily (Practice guideline. Available at https://pubmed.ncbi.nlm.nih.gov/26433807/). PW, Pregnant woman; BM, Breastfeeding mother.

**Table 2:** Global recommendations on IYCN and their counseling focused actions.

Recommendation	Counseling focused actions	Context specific
1. Immediate skin-to-skin contact at birth and initiation of breastfeeding within one hour	1.1 Importance of immediate and uninterrupted skin-to-skin contact	No
	1.2 Avoiding separation of mother and baby	
	1.3 Early initiation of breastfeeding within one hour of birth	
	1.4 Benefits of colostrum	
	1.5 Observe and assist infant self-attachment	
	1.6 Support correct attachment and position	
	1.7 Identifying feeding cues	
2. Exclusive breastfeeding from birth until infant reaches 6 months of age	2.1 Benefits of exclusive breastfeeding	No
	2.2 Correct position and attachment	
	2.3 Responsive breastfeeding	
	2.4 Avoidance of fluids or food other than breastmilk (unless medically indicated)	
	2.5 Management of breastfeeding difficulties	
	2.6 Harmful effects of formula feeding	
	2.7 Risks of using bottles, teats and pacifiers	
	2.8 Manual expression of breastmilk as needed	
	2.9 Breastfeeding small, preterm, sick baby with no medical complication	
3. Timely introduction of complementary feeding	3.1 Importance of timely introduction of complementary foods	No
	3.2 Feeding techniques	
	3.3 Quantity, consistency and frequency of introductory complementary feeding	
	3.4 Responsive feeding	
4. Age-appropriate complementary feeding	4.1 Importance of appropriate complementary feeding	No
	4.2 Age-appropriate quantity, consistency and frequency of feeding	
	4.3 Including locally available diverse foods	
	4.4 Responsive feeding	
	4.5 Handwashing at critical times	
	4.6 Safe preparation, handling and storage of complementary foods	
	4.7 Child feeding during illness	
5. Continued breastfeeding until child is 2 years and beyond	5.1 Importance of continued breastfeeding	No
6. Growth Promotion and Monitoring	6.1 Weight and height/length measurement for all children under 5	No
	6.2 Nutritional counseling for children based on assessment	
	6.3 Management plan for overweight, obese children at primary health care facilities	
7. Assessment and management of wasting	7.1 Screening for severe acute malnutrition (SAM) and counseling (in-patient)	No
	7.2 Screening for SAM and counseling (out-patient)	
	7.3 Screening for moderate acute malnutrition (MAM) and counseling	
8. IFA supplement to children >6 m: 10–12.5 mg elemental iron as syrup/drops daily for three consecutive months if anemia prevalence in infants and young children is ≥40%	8.1 Importance of daily consumption	Yes
	8.2 Continued and consistent consumption	
	8.3 How to take supplements	
	8.4 How to manage side effects	

World leaders, including leaders from South Asia, have committed to strengthening PHC by increasing attention to human resources, knowledge and capacity building, technology advancements, and financing for better health outcomes (27). In 2018–19, governments and development partners from all South Asian countries committed to align national guidance to global recommendations on MIYCN and maximize the use of available platforms to integrate MIYCN services and evidence-informed SBCC, among other consensus action points (28). In keeping with their commitments, governments from South Asian countries are calling for action to strengthen PHC and nutrition services.

UNICEF Regional Office of South Asia (ROSA) and Alive & Thrive, a nutrition initiative managed by FHI Solutions, collaborated to landscape MIYCN counseling in South Asian countries to: (1) ascertain coverage of global counseling recommendations in MIYCN-related policies in South Asian countries, (2) analyze the latest national survey data sets to assess coverage, continuity, intensity, and quality of MIYCN counseling, (3) identify system bottlenecks to deliver quality MIYCN counseling and opportunities to overcome these and (4) curate lessons from country experiences in strengthening MIYCN counseling services.

## Methods

A mixed-methods approach was used to achieve the four objectives listed above. The study duration was eight months from July 2022 to February 2023. Seven of eight South Asian countries—Bangladesh, Bhutan, India, Maldives, Nepal, Pakistan, and Sri Lanka—were included. Afghanistan was excluded as access to key respondents and documents was challenging in the time frame. In both India and Pakistan, two sub-national geographies were closely examined to understand intra-country variations in MIYCN counseling. Sub-national geographies were the states of Bihar and Telangana in India, and the provinces of Punjab and Balochistan in Pakistan. Target groups of interest were pregnant women, recently delivered women and their newborns, mothers or caregivers of children under-two years, and families. Technical scope covered all routine counseling as per global recommendations. At-risk counseling was considered for those affected by undernutrition, including thin women, children with severe and moderate acute malnutrition (SAM/MAM), those affected by overweight, obesity and anemia, and women and children with adolescent pregnancies. Medical complications associated with nutritional risks were excluded. Individual and group counseling along with information dissemination services were included. Mass media was only covered as an enabler. Special campaigns for nutrition verticals like Vitamin A were excluded.

Three sources of information were desk reviews, key informant interviews (KIIs) and the Demographic Health Survey (DHS) in all countries except Bhutan (29–35). In Nepal, DHS released a new report in 2022. As the 2022 data set was not released until the time this study was completed, the earlier version (2016–17) was used for analysis. However, for any published DHS data, the new 2022 report was used. In Bhutan, the National Nutrition Survey was more recent, and thus used for this study (36).

Documents for the desk review were sourced from Ministry websites, PubMed, snowballing, and key informants. The search for research papers was restricted to the last 10 years and published in English. Ninety-three (93) policies and guidelines, 180 counseling materials and over 50 program, research, and data documents were identified. A review of country policies and guidelines was done against the list of 93 global recommendations, disaggregated by recommendations for pregnant women, breastfeeding mothers, and infants and young children. Counseling materials were reviewed against messages extracted from the list of 93 global recommendations. Counseling materials which included pictures or illustrations of people were also reviewed against an eight-point gender and inclusion checklist. The eight points were: (1) equal number of men and women depicted, (2) equal number of boys and girls depicted, (3) images do not enforce stereotypes (e.g., girls/women doing household chores while boys playing, using gadgets), (4) professionals—doctors, nurses, counselors—are either women or both men and women depicted equally, (5) leaders—village head/elected officials—are either women or both men and women depicted equally, (6) use of gender neutral pronouns or he/she, (7) any images of adults/children with disability, and (8) images reflect national diversity.

A total of 115 KIIs were conducted with the support of national staff or consultants in the seven countries. These staff and consultants were selected based on in-depth understanding of country health and nutrition systems and their access to key respondents, especially from national government departments. In each country, the key informants included representatives from government departments engaged with health and nutrition services, development partners, and UNICEF. By country, the breakdown of key informants was as follows: four in Bangladesh, 10 in Bhutan and Sri Lanka, 32 in India and Maldives, 14 in Nepal, and 13 in Pakistan. In Bangladesh, information was also sourced from over 90 interviews undertaken in another UNICEF-supported project in 2020–21 (37). In Bhutan and Maldives, representatives from public health training institutes were also interviewed. Eight domains were identified for information synthesis from desk reviews and key informant interviews. Three domains related to workforce for MIYCN counseling: (1) types of counseling providers and platforms, (2) capacity and motivation of counseling providers, and (3) supervision. The other five were counseling content and materials, information systems, quality improvement, budgets and expenditures, and social-safety nets linked to MIYCN counseling.

A standard set of indicators was necessary to compare survey data from countries in the region. Thus, survey data analysis was structured after mapping indicators relevant for MIYCN counseling coverage and quality in country datasets. Data analysis was done at three levels. At level one, the reach of platforms to potentially deliver MICYN counseling – ANC contacts for pregnancy, institutional delivery, and postnatal contacts for breastfeeding mothers, and any health worker contacts for children six to 23 months – was assessed. Reach was assessed by location (rural vs. urban), wealth index-based quintile classification, access to mobile phones and mass media, and type of service providers (skilled vs. unskilled, public vs. private). At the second level, delivery of counseling services through these platforms was inferred by comparisons with nutrition behaviors. The five nutrition behaviors of interest were: (1) consumption of iron folic acid tablets for 100+ days among those receiving at least four ANC checkups (2) early initiation of breastfeeding among those receiving at least four ANC checkups and institutional delivery, (3) exclusive breastfeeding among those receiving postnatal care (PNC) within 48 h, (4) timely initiation of complementary feeding among those receiving any health worker contacts, and (5) minimum dietary diversity among children receiving any health worker contacts (or not). Finally, five regression models were constructed for each country to determine association of practice of recommended nutrition behaviors with background characteristics of respondents, reach of mass media, ownership of mobile phone, reach of MIYCN contacts, and type of providers. Adjusted effect of these variables, that is background characteristics of respondents and others, on the nutrition behaviors of interest were checked using binary logistic regression analysis. All analysis was done on STATA 15.

## Results

### Country policies on MIYCN counseling

In all seven countries, policies on 18 universally applicable counseling recommendations for pregnant women are available. These recommendations pertain to diet diversity, physical activity, iron folic acid (IFA) compliance, breastfeeding, and hygiene. However, not all are aligned with recent global updates (Table 3). Recommendations on gestational age-specific counseling on diet (and other care) are available in three of seven countries: Bangladesh, India and Maldives. Only one in seven countries, Maldives, has detailed guidance on physical activity in pregnancy and gestational weight gain counseling based on pre-pregnancy body mass index (BMI). The 12 context-specific counseling recommendations in pregnancy have variable policy coverage across countries. Universal food supplementation with counseling is available only in India, and remains needed in two more countries, Bangladesh and Bhutan, where the prevalence of thinness among women is >20%; Sri Lanka follows this recommendation, despite the country’s 9% prevalence of thinness among women. Counseling for management of acute malnutrition among pregnant women is covered in guidance from Bangladesh, Nepal, Pakistan and Sri Lanka. Despite high multiple micronutrient deficiencies among women, none of the countries has policy for antenatal multiple micronutrient supplements (MMS) and counseling to promote its use. It is noted that MMS has only 30 mg iron, half of the recommend levels for antenatal supplementation in countries where anemia is a public health problem. Counseling on calcium supplementation in pregnancy is not included in pregnancy care in Bhutan and Nepal, despite evidence that it is effective in reducing the risk of pre-eclampsia. Policy for counseling on caffeine reduction in pregnancy is unavailable in all countries, except Bangladesh. In India, counseling on caffeine reduction is covered only for women with hypertensive disorder (Table 3).

**Table 3:** Coverage of key recommendations on pregnancy counseling in policy documents (Pol) and programs (Prg), South Asia.

Global nutrition recommendations	Counseling recommendations	Context specific	BGH		BHN		IND		MAL		NPL		PAK		SRL	
			Pol	Prg	Pol	Prg	Pol	Prg	Pol	Prg	Pol	Prg	Pol	Prg	Pol	Prg
Counseling about healthy eating	Consume variety of locally available foods	No														
	Consume fortified foods (iodized salt and others)															
Counseling about keeping physically active	Attain or maintain a healthy weight and /or prevent excessive weight gain	No														
	Take adequate rest															
	Avoid heavy workload															
In undernourished populations, nutrition education on increasing daily energy and protein intake	Increase number of meals using locally available foods	Yes. ≥20% women thin			NA	NA			NA	NA	NA	NA			NA	NA
	Consume food supplement provided through government programs				NA	NA			NA	NA	NA		NA		NA*	NA*
Daily oral iron and folic acid supplementation (30 mg to 60 mg elemental iron, 400 μg folic acid)	Importance of daily consumption	No														
	Continued and consistent consumption															
	How to take supplements															
	How to manage side effects															
Intermittent oral iron and folic acid supplementation with 120 mg of elemental iron and 2,800 μg (2.8 mg) of folic acid once weekly	Dosage and how to consume	Yes. <20% women anemic	NA	NA	NA	NA	NA	NA	NA	NA	NA	NA	NA	NA	NA	NA
	How to manage side effects		NA	NA	NA	NA	NA	NA	NA	NA	NA	NA	NA	NA	NA	NA
Antenatal multiple micronutrient supplement (MMS)	Dosage and how to consume	Yes. Rigorous research		NA		NA		NA		NA		NA		NA		NA
	Continued and consistent consumption			NA		NA		NA		NA		NA		NA		NA
In populations with low dietary calcium intake, daily calcium supplementation (1.5–2.0 g oral elemental calcium)	Importance of daily consumption	Yes diet calcium deficient										NA				
	Continued and consistent consumption											NA				
	How to take supplements											NA				
	How to manage side effects											NA				
Reducing caffeine among pregnant women	Reduce frequency of caffeinated drinks	Yes. Daily intake >300 mg				NA		NA				NA		NA		NA
	Avoid tea/coffee with meals											NA				
Breastfeeding	Importance of breastfeeding	No														
	Immediate and uninterrupted skin-to-skin contact after birth															
	Early initiation of breastfeeding															
	Correct positioning and attachment															
	Exclusive breastfeeding till completion of 6 months of age															
	Responsive breastfeeding															
	Risks of giving formula, breast milk substitutes, using bottles, teats and pacifiers															
Counseling on handwashing at critical times and food hygiene	Handwashing at critical times	No														
	Safe handling, preparation, and storage of food															

Green: Fully aligned to recommendation. Yellow: Partially aligned to recommendation. Red: Not aligned to recommendation. NA, Not Applicable (In reference to policy, implies country context does not support recommendation. In reference to programs, implies lack of policy support). *Sri Lanka does not require universal food supplementation in pregnancy but has policy and program support.

In all countries, policies support 17 of the 19 universally applicable counseling recommendations for breastfeeding mothers (Table 4). The two missed counseling recommendations pertain to type and duration of physical activity in all countries except Maldives. All countries, except Maldives, follow a daily IFA supplementation program, including counseling, to promote IFA supplementation compliance for breastfeeding mothers. The duration of IFA supplementation varies from 30 days post-partum in Pakistan to 180 days in India and Sri Lanka. The coverage of six context-specific counseling recommendations for breastfeeding mothers varies, similar to those for pregnant women (Table 4).

**Table 4:** Coverage of key recommendations on counseling for breastfeeding mothers in policy documents (Pol) and programs (Prg), South Asia.

Global nutrition recommendations	Counseling recommendations	Context specific	BGH		BHN		IND		MAL		NPL		PAK		SRL	
			Pol	Prg	Pol	Prg	Pol	Prg	Pol	Prg	Pol	Prg	Pol	Prg	Pol	Prg
Counseling about healthy eating	Consume variety of locally available foods	No														
	Consume fortified foods (iodized salt and others)															
Counseling about keeping physically active	Limit time spent sedentary	No														
	Do at least 150 min of physical activity throughout the week															
	Incorporate a variety of physical and muscle-strengthening activities; add gentle stretches															
In undernourished populations, nutrition education on increasing daily energy and protein intake	Increase number of meals using locally available foods	Yes. ≥20% women thin			NA	NA			NA	NA	NA	NA			NA	NA
	Consume food supplement provided through government programs					NA			NA	NA	NA		NA		NA*	NA*
Daily oral iron and folic acid supplementation (30 mg to 60 mg elemental iron, 400 μg folic acid)	Importance of daily consumption	No														
	Continued and consistent consumption															
	How to take supplements															
	How to manage side effects															
Intermittent oral iron and folic acid supplementation with 120 mg of elemental iron and 2,800 μg (2.8 mg) of folic acid once weekly	Dosage and how to consume	Yes. <20% women anemic	NA	NA	NA	NA	NA	NA	NA	NA	NA	NA	NA	NA	NA	NA
	How to manage side effects		NA	NA	NA	NA	NA	NA	NA	NA	NA	NA	NA	NA	NA	NA
Reducing caffeine among breastfeeding mothers	Reduce frequency of caffeinated drinks	Yes. Daily intake >300 mg				NA		NA				NA		NA		NA
	Avoid tea/coffee with meals											NA				
Breastfeeding	Importance of breastfeeding	No														
	Immediate and uninterrupted skin-to-skin contact after birth															
	Early initiation of breastfeeding															
	Correct positioning and attachment															
	Exclusive breastfeeding until completion of 6 months of age															
	Responsive breastfeeding															
	Risks of giving formula, breast milk substitutes, using bottles, teats and pacifiers															
Counseling on handwashing at critical times and food hygiene	Handwashing at critical times	No														
	Safe handling, preparation and storage of food															
Dietary advice and information on factors associated with constipation	Consumption of high fiber foods and fluids	No														

Green: Fully aligned to recommendation. Yellow: Partially aligned to recommendation. Red: Not aligned to recommendation. NA: Not Applicable (In reference to policy, implies country context does not support recommendation. In reference to programs, implies lack of policy support). ^*^Sri Lanka does not require universal food supplementation for breastfeeding mothers but has policy and program support.

The 28 universal infant and young child feeding (IYCF) counseling recommendations are covered in policy documents across all seven countries (Table 5). Growth monitoring promotion (GMP) policies for counseling for children at normal and below-normal thresholds for growth are consistently recommended across all seven countries. Other than Maldives and Sri Lanka, none of the countries has policy support for screening and management of overweight and obesity in children under 5. In-facility treatment for SAM is well defined and mostly available; however, outpatient treatment for uncomplicated SAM as well as MAM is sparingly available – most countries offer only counseling to improve diets and hygiene behaviors to manage MAM. Of five countries that require counseling on IFA supplementation for children 6–59 months, namely Bhutan, India, Maldives, Nepal, and Pakistan, only India has a universal program (Table 5).

**Table 5:** Coverage of key recommendations on IYCN counseling in policy documents (Pol) and programs (Prg), South Asia.

Global nutrition recommendations	Counseling recommendations	Context specific	BGH		BHN		IND		MAL		NPL		PAK		SRL	
			Pol	Prg	Pol	Prg	Pol	Prg	Pol	Prg	Pol	Prg	Pol	Prg	Pol	Prg
Immediate skin-to-skin contact at birth and initiation of breastfeeding within 1 h	Importance of immediate and uninterrupted skin-to-skin contact	No														
	Avoid separation of mother and baby															
	Early initiation of breastfeeding (within an hour of birth)															
	Benefits of colostrum															
	Observe and assist infant self-attachment															
	Support correct attachment and position															
	Identifying feeding cues															
Exclusive breastfeeding from birth till infant completes six months of age	Benefits of exclusive breastfeeding	No														
	Correct position and attachment															
	Responsive breastfeeding															
	Not given any other fluid or food other than breastmilk (unless medically indicated)															
	Management of breastfeeding difficulties															
	Harmful effects of formula feeding															
	Risk of using bottles, teats and pacifiers															
	Manual expression of breast milk as needed															
	Breastfeeding small, preterm, sick baby without medical complication															
Timely introduction of complementary feeding	Importance of timely introduction of complementary feeding	No														
	Feeding techniques															
	Quantity, consistency and frequency of introductory complementary feeding															
Age appropriate complementary feeding	Responsive feeding															
	Importance of appropriate complementary feeding															
	Age-appropriate quantity, consistency and frequency of feeding	No														
	Including locally available diverse foods															
	Responsive feeding															
	Handwashing at critical times															
	Safe preparation, handling and storage of complementary foods															
	Child feeding during illness															
Continued breastfeeding till child is two years and beyond	Importance of continued breastfeeding	No														
Growth Monitoring Promotion	Weight and height/length measurement for all children under-5	No														
	Nutritional counseling for children under-5 based on assessment															
	Management plan for overweight and obese children under-5 at primary health care facilities															
Assessment and management of wasting	Screening for SAM and counseling (in-patient)	No														
	Screening for SAM and counseling (out-patient)															
	Screening for MAM and counseling (community)															
IFA supplement to young children >6 m: 10–12.5 mg elemental iron as syrup/drops daily for 3 consecutive months	Importance of daily consumption	Yes (anemia ≥40% in infants & young children)	NA	NA											NA	NA
	Continued and consistent consumption		NA	NA											NA	NA
	How to take supplements		NA	NA											NA	NA
	How to manage side effects		NA	NA											NA	NA

Green: Fully aligned to recommendation. Yellow: Partially aligned to recommendation. Red: Not aligned to recommendation. NA: Not Applicable (In reference to policy, implies country context does not support recommendation. In reference to programs, implies lack of policy support).

### Coverage, continuity, intensity, and quality of MIYCN counseling

**Figure 1:**
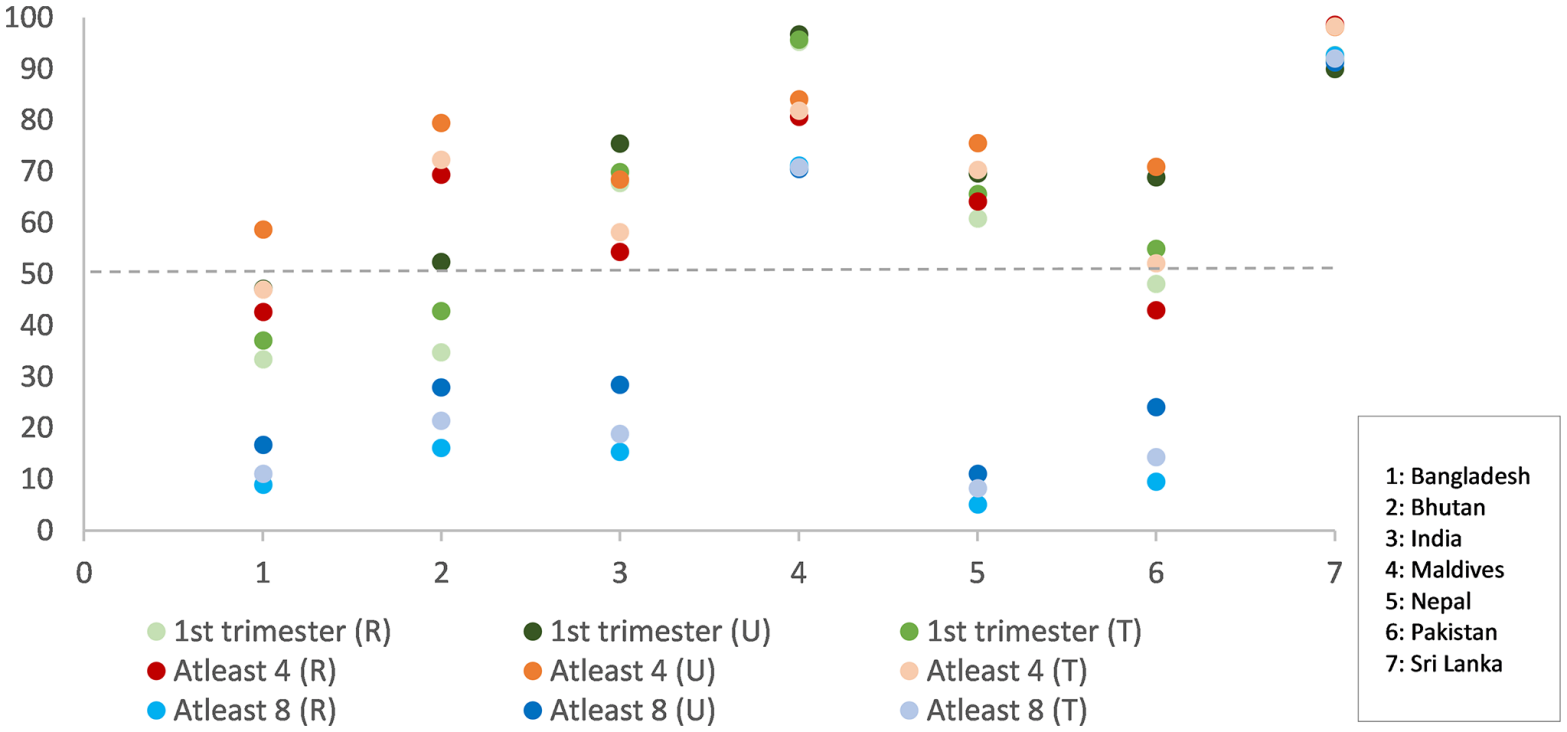
ANC in first trimester, women receiving at least four (4) ANC contacts and women receiving at least eight (8) ANC contacts, total contacts (T), and contacts disaggregated by rural (R)- urban (U) for South Asian countries (in percentages).

The proportion of women who report initiation of ANC within first trimester ranged from 37% (Bangladesh) to 96% (Maldives) (Figure 1) (29–32, 34–36). Five to six of every 10 pregnant women in Bangladesh, Bhutan, and Pakistan did not initiate ANC within the first trimester, a critical time to manage pregnancy symptoms, modify diets, and consume essential nutritional supplements like folic acid, with or without iron. In sub-national geographies of India, half the women in Bihar missed early pregnancy ANC contact. Telangana did much better, with only one in 10 pregnant women missing out on ANC in the first trimester (30). Published data on first trimester ANC contacts were unavailable for the Punjab and Balochistan provinces in Pakistan.

Coverage of at least four ANC contacts ranged from 47% (Bangladesh) to 98% (Sri Lanka) (29–32, 34–36). The proportion of women who reported the receipt of four ANC contacts was lower than those reporting ANC within first trimester for India, Maldives, and Pakistan. About half the pregnant women in Bangladesh, India, and Pakistan did not have at least four ANC contacts, which would be ideal to reinforce recommended antenatal nutrition behaviors. Further, coverage of WHO- and UNICEF-endorsed eight ANC contacts was low in all countries, except Sri Lanka. More than eight in 10 women had a consultation with a skilled health care provider in public or private sector at least once in pregnancy.

At least four ANC contacts was the most consistent and strongest influencer on consumption of 100+ IFA across all countries (Odds Ratios [OR] Bangladesh 3.4, Bhutan 25.2, India 2.3, Maldives 2.7, Nepal 4.8, Pakistan 4.6, Sri Lanka 7) (Table 6). However, in Bangladesh, India, Maldives, and Pakistan, at least 50% of the women who received at least four ANC contacts did not consume 100+ IFA, which suggests opportunities to improve counseling and possibly supply chains.

**Table 6:** Odds of consuming 100+ IFA tablets in pregnancy by background characteristics, receipt of at least four ANC contacts, and women’s access to mid-mass media.

*N*	BGH		BHN		IND		MAL		NPL		PAK		SL	
	5,012	95% CI	887	95% CI	122,458	95% CI	1,693	95% CI	2,761	95% CI	6,276	95% CI	4,635	95% CI
Rural vs. Urban	1.1	0.9–1.3	1.3	0.4–4.9	1.4***	1.3–1.4	NA		0.9	0.7–1.1	1.4***	1.2–1.8	1.1	0.6–2.0
Age < 20 yrs. vs. > =20 yrs	1.1	0.9–1.3	1.7	0.7–4.3	1.0	0.9–1.0			1.4**	1.0–1.9	1.4	0.6–1.3	0.3	0.0–2.2
Wealth quintile poorest *vs*														
poorer	0.9	0.7–1.1	0.8	0.3–2.1	1.2***	1.1–1.2	0.8	0.5–1.3	1.0	0.7–1.3	1.1	0.8–1.6	1.2	0.7–2.1
middle	1.2	0.9–1.5	1.3	0.4–4.6	1.2***	1.2–1.3	0.7	0.4–1.1	1.0	0.7–1.5	1.0	0.7–1.4	1.4	0.7–2.5
richer	1.3**	1.0–1.6	1.5	0.3–7.7	1.4***	1.3–1.5	0.7	0.4–1.2	0.8	0.6–1.2	1.4**	1.0–2.0	1.7*	1.0–3.3
richest	1.9***	1.5–2.5	0.8	0.2–3.4	1.7***	1.6–1.8	1.0	0.6–1.6	1.2	0.8–1.8	2.3***	1.6–3.3	1.3	0.7–2.5
At least 4ANC = yes	3.4**	2.9–3.9	25.2***	12.5–50.6	2.3***	2.2–2.4	2.7***	1.7–4.2	4.8***	3.8–6.0	4.6***	3.6–6.0	7.0***	3.6–13.5
Public vs. private sector provider	0.9	0.8–1.1	NA		0.8***	0.8–0.9	1.2	0.8–1.8	0.7**	0.5–0.9	1.4***	1.1–1.8	1.6**	1.1–2.4
Skilled vs. unskilled health care provider	0.8*	0.6–1.10	NA		0.8***	0.8–0.9	NA		0.6	0.1–2.7	0.3	0.1–1.4	0.2	0.1–0.4
Own mobile phone = yes	1.1	1.0–1.3	NA		1.0	1.0–1.1	1.3	0.5–3.1	1.5***	1.2–1.9	1.0	0.8–1.3	1.1	0.7–1.7
Read newspaper at least once a week = yes	2.0***	1.3–3.1	NA		1.1*	1.0–1.1	1.2	1.0–2.2	2.6**	1.2–5.6	1.6**	1.1–2.5	1.6**	1.0–2.4
Listen to radio at least once a week = yes	1.6**	1.0–2.5	NA		0.8*	0.7–0.8	0.8	0.6–1.4	1.6***	1.3–2.1	0.9	0.6–1.5	1.1	0.8–1.6
Watch television at least once a week = yes	0.9	0.8–1.1	NA		1.4***	1.3–1.4	1.6**	1.0–2.4	1.2	0.9–1.6	1.0	0.8–1.2	1.8***	1.2–2.7
Total number of influencers	6		1		11		2		6		6		5	

*p < 0.1, **p < 0.05, ***p < 0.01. NA, Not available. ^#^Atleast one ANC. Grey: Insufficient sample size.

Compared to 100+ IFA consumption, the reported practice of early initiation of breastfeeding was less influenced by receipt of at least four ANC contacts (Supplementary Table S1).

Positive correlation between early initiation of breastfeeding with number of ANC contacts was noted only in three countries: Bhutan, India, and Maldives (OR 4.9, 1.4, 1.6, respectively). Receiving ANC from private sector provider reduced odds of early initiation of breastfeeding in India (OR 0.9) and Pakistan (OR 0.7). Further, delivering at a private health facility had a negative influence on early initiation of breastfeeding compared with delivering at a public health facility in Bangladesh, India and Nepal (OR 0.7, 0.8 and 0.8, respectively). In addition to counseling in ANC contacts, promotion of early initiation of breastfeeding requires support and counseling immediately after birth.

**Figure 2:**
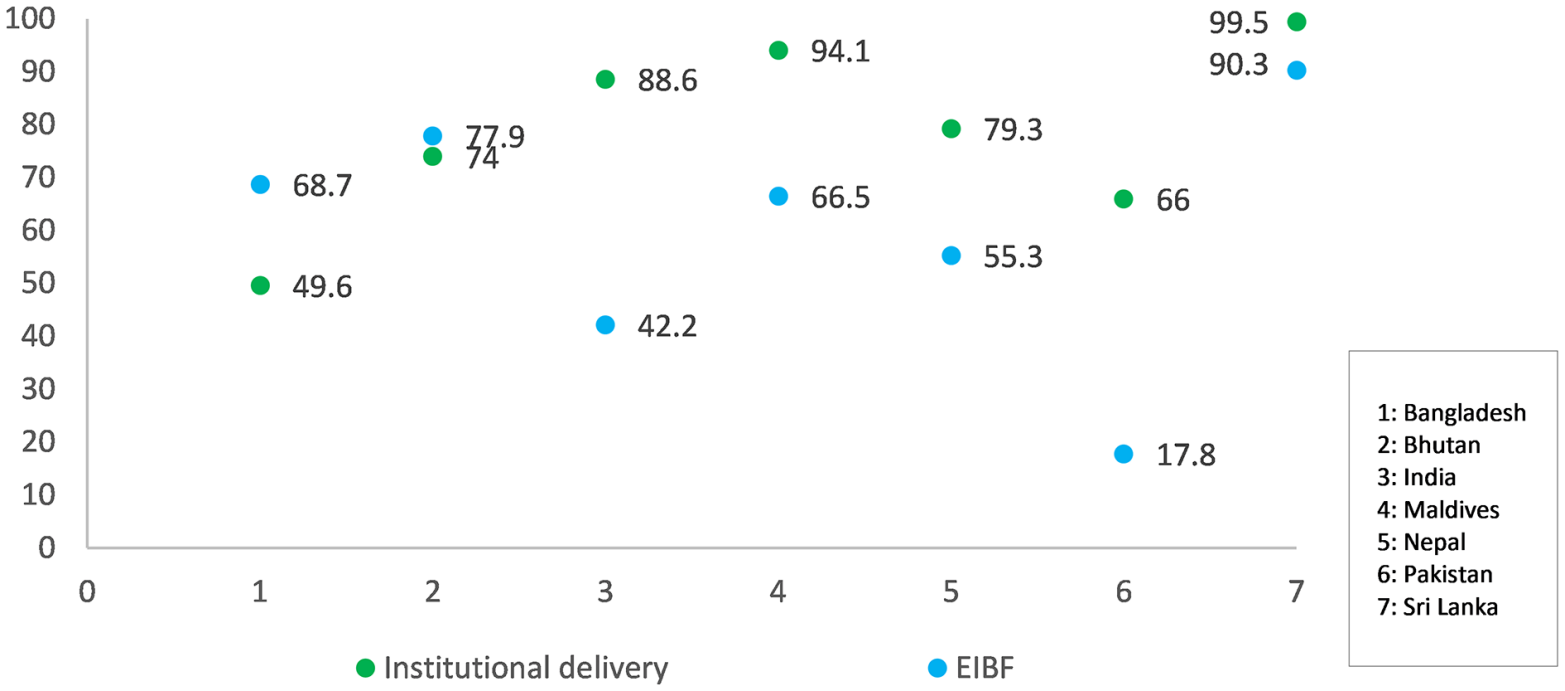
Institutional delivery rates versus early initiation of breastfeeding rates for South Asian countries. Data for early initiation of breastfeeding among those who delivered in a health facility was available for India and Pakistan; for other countries, overall rates were considered.

The widest gap in early initiation of breastfeeding and institutional delivery was noted in Pakistan (48% points) followed closely by India (46% points). In contrast, Bangladesh and Balochistan province of Pakistan had higher proportions of early initiation of breastfeeding than institutional deliveries (Figure 2).

Rates of PNC within 4 hours of birth, which is crucial to establish breastfeeding, were lower than the institutional delivery rates in all countries for which data were available – India, Maldives, Pakistan, and Sri Lanka (29–31, 35) (Supplementary Table S2).

PNC contact within 48 h of birth was positively correlated with exclusive breastfeeding among children under 6 months in India (OR 1.4), Pakistan (OR 3.1) and Sri Lanka (OR 3.2) (Table 7).

**Table 7:** Odds of exclusive breastfeeding by background characteristics, receipt of PNC within 48 h of birth, and women’s access to mid-mass media.

*N*	BGH		BHN		IND		MAL		NPL		PAK		SL	
	5,012	95% CI	887	95% CI	122,458	95% CI	1,693	95% CI	2,761	95% CI	6,276	95% CI	4,635	95% CI
Rural vs. Urban	0.8	0.6–1.1	1.3	0.1–13.5	0.8***	0.7–0.8	NA		0.7	0.5–1.2	0.8	0.5–1.1	1.2	0.6–2.6
Age < 20 yrs. vs. > =20 yrs	1.1	0.8–1.6			0.8**	0.7–1.0			1.0	0.6–1.7	1.4	0.8–2.5	0.8	0.2–4.1
Wealth quintile poorest *vs*														
Poorer	1.2	0.8–1.9			0.9**	0.8–1.0	0.8	0.3–2.1	0.7	0.3–1.4	1.0	0.6–1.7	1.3	0.5–3.5
Middle	1.1	0.6–1.8			0.9**	0.8–1.0	0.5	0.2–1.4	1.1	0.5–2.4	0.9	0.6–1.6	1.5	0.6–3.6
Richer	1.2	0.7–2.0			0.9	0.8–1.0	0.9	0.2–2.0	0.7	0.3–1.5	0.8	0.5–1.4	1.8	0.7–4.9
Richest	1.2	0.7–2.1			0.8***	0.7–0.9	0.6	0.3–2.4	0.8	0.3–2.0	0.7	0.4–1.2	1.3	0.5–3.5
Received PNC <48 h = yes	0.6	0.3–1.5			1.4***	1.2–1.6	0.7	0.3–1.9	1.3	0.2–7.5	3.2*	1.0–10.3	3.1*	1.0–10.7
Public vs. private sector provider	0.9	05–1.4			0.9***	0.8–0.9	1.3	0.5–3.4	0.8	0.4–1.9	1.1	0.7–1.7	0.5	0.2–1.4
Skilled vs. unskilled health care provider	0.7	0.4–1.1			1.1***	1.0–1.2	NA		0.8	0.4–1.6	1.4*	1.0–2.2	NA	
Own mobile phone = yes	0.9	0.7–1.3	NA		1.1*	1.0–1.3			0.8	0.5–1.5	0.9	0.6–1.3	0.6	0.2–1.8
Read newspaper at least once a week = yes	2.1	0.8–5.8	NA		1.0	0.9–1.2	1.2	0.6–2.5	1.2	0.3–4.3	2.1*	1.0–4.5	0.4**	0.2–0.8
Listen to radio at least once a week = yes	0.4	0.1–1.2	NA		0.9	0.7–1.1	0.7	0.3–1.4	1.6	0.9–2.8	1.2	0.6–2.4	0.9	0.5–1.7
Watch television at least once a week = yes	1.0	0.7–1.4	NA		1.1**	1.0–1.2	0.7	0.2–1.8	1.0	0.6–1.7	1.0	0.7–1.4	0.5*	0.2–1.0
Total number of influencers	0		0		10		0		0		3		3	

*p < 0.1, **p < 0.05, ***p < 0.01. NA, Not available. ^#^Atleast one ANC. Grey: Insufficient sample size.

**Figure 3:**
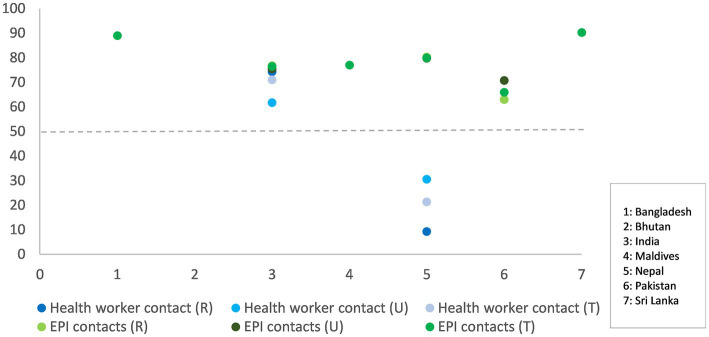
Any health worker contacts versus EPI contacts for children under 24 months, total contacts (T) and contacts disaggregated by rural (R)- urban (U) for South Asian countries. Health worker contact data is available only for India and Nepal.

Data on health worker contacts for infants and young children to promote appropriate feeding practices are available for only two of the seven countries: India and Nepal. In India the data reflect recall of contacts with any skilled or unskilled provider 3 months before the survey but not specifically for nutrition counseling. However, as India has home-based contacts for nutrition counseling and there are monthly GMP sessions, the data potentially represent nutrition counseling contacts. In Nepal, the data reflect recall of contacts specifically on IYCF counseling one year before the survey. The average number of contacts per child were 2.9 for both India and Nepal, but the reference periods were three and 12 months, respectively. In India, data were available on contacts for GMP, specifically weight measurement and counseling post weight measurement. About 62% of children under two were weighed in the 12 months preceding the survey, and 75% of their mothers/caregivers received counseling. Data on all basic vaccination covered by the time a child completes 2 years of age indicate these children were reached at least five times in that time period; most of these contacts occur in the first year of life. Coverage of all basic vaccination (Expanded Program for Immunization [EPI] contacts) is higher than 75% for all countries, except Pakistan where it is 66% (Figure 3). Due to its high and assured service reach, there is an opportunity to layer child nutrition services to EPI platforms in all countries.

Health worker contacts were positively correlated with children six-23 months receiving minimum diversity diet or foods from at least four different food groups in India (OR 1.3). However, the cost of food is a consideration with children belonging to richest wealth quintile faring better on minimum dietary diversity than others in both India and Nepal (Table 8).

**Table 8:** Odds of infants and children 6–24 months receiving minimum diverse diet by background characteristics, receipt of health worker contact, and mother’s access to mid-mass media.

*N*	BGH	BHN	IND		MAL	NPL		PAK	SL
	3,490		84,579	95% CI		2,761	95% CI		3,008
Rural vs. Urban	NA	NA	1.2***	1.1–1.2	NA	1.0	0.8–1.3	NA	NA
Age < 20 yrs. vs. > =20 yrs	NA	NA	1.1	1.0–1.2	NA	1.0	0.7–1.5	NA	NA
Wealth quintile poorest *vs*									
Poorer	NA	NA	1.0	0.9–1.1	NA	1.0	0.6–1.4	NA	NA
Middle	NA	NA	1.1*	1.0–1.2	NA	0.9	0.6–1.4	NA	NA
Richer	NA	NA	1.1***	1.0–1.2	NA	1.8***	1.2–2.8	NA	NA
Richest	NA	NA	1.2***	1.0–1.3	NA	1.9**	1.1–3.1	NA	NA
Contacted by health care provider = yes	NA	NA	1.3***	1.2–1.4	NA	1.0	0.8–1.4	NA	NA
Public vs. private sector provider	NA	NA	1.0	0.9–1.0	NA	1.0	0.6–1.5	NA	NA
Skilled vs. unskilled health care provider	NA	NA	0.9*	0.8–0.9	NA	1.0	0.7–1.5	NA	NA
Own mobile phone = yes	NA	NA	1.0	0.9–1.1	NA	1.4***	1.0–1.8	NA	NA
Read newspaper at least once a week = yes	NA	NA	1.2*	1.1–1.3	NA	2.8***	1.4–5.8	NA	NA
Listen to radio at least once a week = yes	NA	NA	1.5***	1.4–1.8	NA	1.3	0.9–1.7	NA	NA
Watch television at least once a week = yes	NA	NA	1.1*	1.0–1.1	NA	1.3*	1.0–1.7	NA	NA
Total number of influencers			9			5			

*p < 0.1, **p < 0.05, ***p < 0.01. NA, Not available. Blank: Non-significant association.

### Workforce for MIYCN counseling

All South Asian countries except Maldives have a network of community-based health workers with MIYCN counseling roles. In all countries except Bhutan, the workers are either salaried or incentivized for service delivery. MIYCN counseling is an add-on role to traditional roles of family planning, ANC, newborn care, and immunization services for community-based health workers. India is the only country to also have dedicated early child development and nutrition workers. Most countries are affected by a shortage of government supported community-based health workers. The situation is most severe in Bangladesh, with a shortfall of over 20% for all cadres of community health workers (37). However, Bangladesh has over 100,000 non-government supported community workers active in both rural and urban areas. There is a wide range in entry-level capacity of community-based workers. In Bhutan, there is a training requirement of 7 days, while in Pakistan and India, there are certification courses lasting 15 to 18 months. Thus, pre- and in- service training and supervision support requirements need to be contextualized, and the complexity of tasks in job descriptions should be assessed against qualifications (Table 9).

**Table 9:** Selected features of MIYCN counseling providers under the PHC programs, across South Asia.

	Bangladesh		Bhutan	India			Maldives	Nepal	Pakistan	Sri Lanka
Community and outreach										
CHW expected to counsel on nutrition	FWA	HA	VHW	AWW	ASHA	ANM (at subcenter)	NA	FCHV	LHW	PHM
Nutrition counseling role in job description	Yes	Partial	Yes	Yes	Yes	Yes	NA	Yes	Yes	Yes
Ideal population norm (Rural)	1: 4,500	1:6,000	1:250	1:400–800 ^#^	1:1,000	1:5,000	NA	1:150–1,000	1:1,000–1,500	1:3,000
Actual population ratio (Rural)	1: 8,324	1: 10,570	1:372	1:780	1:1,030	1:5,000	NA	1:509	1:1,789*	1:3,876*
Total number (Rural)	20,908	15,420	1,190	1,166,617	883,062	187,557	NA	46,088	92,849*	5,716*
CHW available in urban areas	No	No	No	Yes	Yes	(At PHC)	NA	Yes	Yes	Yes
Ideal population norm	NA	NA	NA	1:800	1:1,000–2,500		NA	1:500–1,000	1:1,500 (slums)	1:3,000
Actual population norm				-	-	-	NA	1:1,170	-	-
Total number	NA	NA	NA	136,000	56,916	20,937 (at PHC)	NA	5,328	-	-
Duration of pre-service training	21 days	21 days	7 days	26 days	8 + 20 days	18 + 6 months	NA	18 days	15 months	6 months
In-service training on nutrition counseling	Yes	Yes	No	Yes	Yes	No	NA	Yes	Yes	Yes
Supervisory structure defined	Yes (FPI)	Yes (AHI/HI)	Yes (Health Assistants)	Yes (Anganwadi supervisor)	Yes (ASHA facilitator)	Yes (Sr. Nurse or MO)	NA	Yes (AHW/ANM)	Yes (LHS)	Yes (Supervising PHM)
PHC facilities										
Cadre expected to counsel on nutrition	FWV	CHCP, SAM management unit staff	Health Assistants	NA	NA	ANM, SN, MO	ACHO, CHO, FHO	AHW, ANM, Sr. nurses, MO, HA	LHS, WMO SAM management unit staff	PHN, MO
Receive in-service nutrition training	No	Yes	No	-	-	Yes	Yes (IYCF only)	Yes (AHW, ANM, HA)	Yes	Yes
Cover urban areas	No	No	Yes	Yes	Yes	Yes	Yes	Yes	Yes	Yes

^#^1: 400 for mini Anganwadi and 1:800 for Anganwadi in rural or urban. In hard to reach areas norm is 1: 300–800 for Anganwadi and 1: 150–300 for mini Anganwadi. ^*^Numbers of community-based workers are total for rural and urban. Actual population coverage cannot be estimated due to lack of disaggregated rural–urban numbers.

Quality of trainings is a concern. In Balochistan, Pakistan, annual refresher trainings for Lady Health Workers are stalled due to lack of funds. Evidence from Bihar, India indicates that despite high coverage of incremental learning among *Anganwadi* Workers (~ 90% trained) and organization of community-based events (events hosted in ~80% community sites), coverage has not translated to expected changes in nutrition behaviors at the household level (38). The qualifying trainings for community-based health workers, including skilled workers, include some nutrition components but are universally weak on maternal nutrition counseling. Also, trainings across the region have limited content on gender sensitization and negotiation skills. Key informants in Bangladesh and India stated that female community health workers face the same socio-cultural and gender barriers as other women in the community. They need training on negotiating nutrition behavior changes with family members of pregnant women and children. Key informants also stated that even when SBCC resources were available, their use by both skilled and unskilled workers is not guaranteed. Reasons for this include a lack of standard protocols on use of these resources at specific contact points, lack of training on how to use the resources, and that the resources are not attractive enough for the clients or not contextualized. Service providers get better reception when counseling is linked to a commodity or any takeaway for the clients; this also affects workers’ motivation to counsel. Key informants from all countries noted that more skilled workers, like doctors and nurses, invariably focus on curative services and less on counseling. Graduate training courses for doctors and nurses have limited theory and practical sessions on nutrition (39). This was corroborated by key informants from Bangladesh, India, Nepal and Maldives.

The short contact time with clients and lack of privacy at outreach sites are severe limitations in delivering MIYCN counseling services (40). In settings where home visits or community group meetings are also unlikely, such as in areas that are hard to reach, highly conservative, and/or experiencing a severe shortage of frontline workers, there is practically no opportunity for face-to-face interaction; thus, options for technology-enabled MIYCN messaging and mass media become very relevant. There are no dedicated platforms for male engagement in MIYCN counseling, except in Sri Lanka. Most supervisors are constrained to travel to all planned sites due to limited transport allowances but there is opportunity to prioritize visits and devise methods of group supervision.

### MIYCN counseling content and materials

In most countries, one to two departments within the Ministry of Health lead development of MIYCN counseling materials. Donor agencies and development partners are engaged in development of these materials. Seventy (70) of the 96 (73%) materials for pregnant women, 60 of the 86 (70%) for breastfeeding mothers and 69 of 95 (73%) for mother/caregivers of children six to 23 months were in paper format. Under 15% of these were materials for clients to take home. With increasing access to technology, web-based information and audio-visual content is gaining relevance and reach. Technology based solutions directly for clients were noted in Maldives and in experimental stage in Bihar, India. In Maldives, nutrition information for first 1,000 days is available through *Yagooth* mobile application. In Bihar an application based game *Chatkaarey* B enables users to access information on family nutrition. Both applications are available through Google Play Store. In Nepal, series of engaging videos on first 1,000 days nutrition with special focus on male engagement were developed under the *Banchin Ama* (Mother says) campaign. Video campaigns are also available through various national programs in Bangladesh, Bhutan, India, Maldives and Pakistan (Table 10).

**Table 10:** Review of counseling materials relevant for pregnant women (T = Total).

		BGH	BHN	IND	MAL	NPL	PAK	SRL	T
Total number of materials reviewed		23	13	41	6	4	5	4	96
Print materials		**16**	**4**	**33**	**5**	**3**	**5**	**4**	**70**
*for* Display, demonstration		11	4	32	3	3	2	4	59
*as* Take aways		05	0	1	2	0	3	0	11
Videos and other e-materials		**7**	**9**	**8**	**1**	**1**	**0**	**0**	**26**
Themes		Number of materials							
Healthy eating	Consume variety of locally available foods	19	2	33	4	3	0	2	63
	Consume fortified foods (iodized salt and others)	1	0	25	3	0	0	1	30
Keeping physically active	Attain or maintain a healthy weight and/or prevent excessive weight gain	6	3	8	2*	2	0	0	21
	Take adequate rest	5	1	10	2	2	0	1	21
	Avoid heavy workload	3	0	8	0	0	0	1	12
In undernourished populations, increasing daily energy and protein intake	Increase number of meals using locally available foods (Special reference to thin women)	0	NA	17	NA	NA	NA	NA	17
	Consume food supplement provided through government programs	0	NA	8	NA	NA	NA	1	9
Daily oral iron and folic acid supplementation	Importance of daily consumption	7	1	19	3	2	1	1	34
	How to take supplements	3	1	18	2	2	0	0	28
	How to manage side effects	1	0	14	2	0	0	0	17
In populations with low dietary calcium intake, daily calcium supplementation	Importance of daily consumption	4	1	10	NA	0	0	1	16
	How to take supplements	4	1	8	NA	0	0	0	13
Reducing caffeine intake	Reduce frequency of caffeinated drinks	1	0	6	2	0	0	0	9
	Avoid tea/coffee with meals	2	1	13	2	0	0	0	18
Breastfeeding	Importance of breastfeeding	13	4	21	3	3	3	1	47
	Immediate and uninterrupted skin-to-skin contact after birth	5	2	10	3	3	3	1	27
	Early initiation of breastfeeding	13	2	21	3	3	3	1	46
	Correct positioning and attachment	5	3	10	3	2	3	1	27
	Exclusive breastfeeding till completion of 6 months of age	13	4	17	3	3	3	1	44
	Responsive breastfeeding	2	2	5	3	2	3	1	18
	Risks of giving formula, breast milk substitutes, using bottles, teats and pacifiers	3	2	12	0	0	0	0	17
Counseling on handwashing, food hygiene	Handwashing at critical times	4	0	9	1	2	2	0	18
Messages customized to gestational age		2	0	4	1	0	0	0	7

*MAL- Gestational weight gain counseling as per IOM BMI based classification.There were two types of materials reviewed-Printed (paper format) and videos/e materials. Under printed materials there were two sub-categorires-Dispay and take away. Values against two main types of materials are bold to distinguish from the sub-categories.

Maternal nutrition messages for pregnant women and breastfeeding mothers mostly relate to healthy eating, specifically, eating locally available diverse foods; 66% of materials for pregnant women and 49% for breastfeeding mothers carried these messages. However, physical activity, weight gain and rest in pregnancy, and consumption of IFA and calcium supplements are much less covered (9 to 21 of 96 materials reviewed). An innovation from Telangana, India is inclusion of a page with 180 dots in the Mother and Child card to enable pregnant women to track daily IFA tablet consumption. Messaging on gestational weight gain in most countries (except Maldives) follows standard 10–12 kg weight gain recommendation without consideration of pre-pregnancy BMI or other nutritional status parameters for pregnant women, like mid-upper arm circumference. Gestational age-specific messaging is missing across all countries except Maldives and is present in some materials from India and Bangladesh (Tables 11, 12).

**Table 11:** Review of counseling materials relevant for breastfeeding mothers (T = Total).

		BGH	BHN	IND	MAL	NPL	PAK	SRL	T
Total number of materials reviewed		16	17	35	4	3	8	3	86
Print materials		**11**	**6**	**27**	**3**	**2**	**8**	**3**	**60**
*for* Display, demonstration		8	6	26	3	2	2	3	50
*as* Take aways		3	0	1	0	0	6	0	10
Videos and other e-materials		**5**	**11**	**8**	**1**	**1**	**0**	**0**	**26**
Themes									
Healthy eating	Consume variety of locally available foods	11	3	21	3	3	0	1	42
	Consume fortified foods (iodized salt and others)	1	0	15	3	1	0	0	20
Keeping physically active	Limit time spent sedentary	0	1	0	2	0	0	0	3
	Do at least 150 min of physical activity throughout the week	0	0	0	1*	0	0	0	1
In undernourished populations, increasing daily energy and protein intake	Increase number of meals using locally available foods	0	NA	8	NA	NA	NA	NA	8
	Consume food supplement provided through government programs	0	NA	6	NA	NA	NA	1	7
Daily oral iron and folic acid supplementation	Importance of daily consumption	4	1	15	NA	2	1	0	23
	How to take supplements	2	1	16	NA	2	0	0	20
	How to manage side effects	0	0	6	NA	1	0	0	7
Reducing caffeine intake	Reduce frequency of caffeinated drinks	0	0	0	2	0	0	0	2
	Avoid tea/coffee with meals	0	1	8	2	0	0	0	11
Breastfeeding	Importance of breastfeeding	6	4	22	3	4	6	1	46
	Immediate and uninterrupted skin-to-skin contact after birth	4	2	11	3	4	4	1	29
	Early initiation of breastfeeding	6	2	20	3	4	4	1	40
	Correct positioning and attachment	5	3	12	3	4	3	1	31
	Exclusive breastfeeding until 6 months of age	6	4	21	3	4	4	1	43
	Responsive breastfeeding	1	2	15	3	4	3	1	29
	Overcoming problems in breastfeeding	3	0	4	1	1	0	0	9
	Risks of giving formula, breast milk substitutes, using bottles, teats and pacifiers	2	2	17	0	1	0	1	23
Counseling on handwashing and food hygiene	Handwashing at critical times	3	0	11	1	2	2	0	19
Dietary advice and information on factors associated with constipation	Consumption of high fiber foods and fluids	1	0	4	1	0	0	0	6

^*^MAL Duration of activity as 150 min per week specified for all adults.There were two types of materials reviewed-Printed (paper format) and videos/e materials. Under printed materials there were two sub-categorires-Dispay and take away. Values against two main types of materials are bold to distinguish from the sub-categories.

**Table 12:** Review of counseling materials relevant for mothers of children 6–23 months (T = Total).

		BGH	BHN	IND	MAL	NPL	PAK	SRL	T
Total number of materials reviewed		19	17	28	7	6	8	10	95
Print materials		**14**	**6**	**23**	**6**	**3**	**8**	**9**	**69**
*for* Display, demonstration		11	6	22	5	3	3	8	58
*as* Take aways		3	0	1	1	0	5	1	11
Videos and other e-materials		**5**	**11**	**5**	**1**	**3**	**0**	**1**	**26**
Themes									
Timely introduction of complementary feeding	Importance of timely introduction of complementary feeding	10	4	20	3	5	2	5	48
	Feeding techniques	6	4	16	3	5	1	5	40
	Quantity, consistency and frequency of introductory complementary feeding	7	4	16	3	5	1	5	41
Age appropriate complementary feeding	Importance of appropriate complementary feeding	7	4	20	3	5	1	5	45
	Age-appropriate quantity, consistency and frequency of feeding	7	4	20	3	5	1	5	45
	Including locally available diverse foods	9	10	21	5	5	6	6	62
	Responsive feeding	6	4	16	3	3	1	5	38
	Handwashing at critical times	5	11	17	3	5	2	1	44
	Safe preparation, handling and storage of complementary foods	4	11	17	3	4	1	1	41
	Child feeding during illness	4	2	17	2	3	1	1	30
Continued breastfeeding till child is two years and beyond	Continued breastfeeding	5	2	19	2	4	1	5	38
Growth Monitoring Promotion	Nutritional counseling for children under-5 based on assessment	2	0	9	2	2	0	1	16
	Management plan for overweight and obese children under-5 at primary health care facilities	0	0	0	0	0	0	0	0
Assessment and management of wasting	Explaining GMP	3	0	6	2	2	0	1	14
IFA supplement to young children >6 m*	Importance of daily consumption	NA	NA	10	NA	NA	NA	NA	10
	Continued and consistent consumption	NA	NA	10	NA	NA	NA	NA	10
	How to take supplements	NA	NA	10	NA	NA	NA	NA	10
	How to manage side effects	NA	NA	4	NA	NA	NA	NA	4

*Only India has universal IFA supplementation program for this age group.There were two types of materials reviewed-Printed (paper format) and videos/e materials. Under printed materials there were two sub-categorires-Dispay and take away. Values against two main types of materials are bold to distinguish from the sub-categories.

Standard messages on breastfeeding, specifically early initiation of breastfeeding and exclusive breastfeeding, are well covered in most countries and in materials for all target groups. However, fewer materials provide information on correct positioning and responsive feeding. Complementary feeding messages on timely initiation and age-appropriateness had better coverage than responsive feeding and feeding during illness. Age-appropriate complementary feeding messages covered feeding quantity, frequency, consistency, and diversity of foods for infants 6–8 months, 9–11 months, and 12 to 23 months. However, messages on importance of GMP and nutritional assessment-based counseling were limited to 17% of the 95 materials for children six-23 months. Messages on prevention and guidance on management of nutritional risks for all target groups had limited coverage across countries (Tables 11, 12).

Counseling materials from five of the seven countries – Bangladesh, India, Maldives, Nepal and Sri Lanka – depicted men caring for pregnant women, mothers, and children, did not enforce stereotypical roles, and showed women as skilled care and counseling providers (Supplementary Table S3). Except India, MIYCN counseling materials targeting local leaders as influencers were lacking. Most printed counseling materials from all countries, except Maldives and Sri Lanka, predominantly focused on rural audiences. No MIYCN counseling materials contextualized for urban settings, including the more vulnerable urban poor, were identified in these countries.

### Information systems for MIYCN counseling

All countries include indicators on ANC, institutional deliveries, and PNC contact points that have the potential to deliver MIYCN counseling services. However, contact points for infants and children are reported in fewer countries, namely, Bangladesh, India, Maldives, and Sri Lanka. Key informants raised concerns on the quality of reporting in all countries, except Sri Lanka. Coverage of all MIYCN counseling indicators is limited, with only four of the seven countries – Bangladesh, India, Maldives, and Sri Lanka – having data on counseling services for maternal nutrition, breastfeeding, and GMP. In these four countries, counseling contacts, such as number of antenatal classes and GMP sessions in Sri Lanka and outreach fixed-day services and home visits organized by health and nutrition workers in India, are tracked. However, only in India and partially in Bangladesh, are these data available disaggregated by provider to assess service reach of community-based versus facility-based providers. Individual client tracking is possible in both Bangladesh and India. In Bangladesh, clients registered at community clinics can be tracked, while in India, individual tracking is possible for clients registered under the national nutrition program, Integrated Child Development Services (ICDS), through the Poshan Tracker, as well as for those registered for ANC in the reproductive child health portal. In Telangana, India, the Nutrition and Health Tracking System has been in operation since 2016 to monitor receipt of services by every registered pregnant woman, breastfeeding mother and child under-six and reaching at-risk cases timely with required services. Bangladesh is the only country to have reporting on both coverage and quality of MIYCN counseling through the World Bank-supported Disbursement Linked Indicators (DLI) reporting. Two counseling indicators are covered, namely (1) any nutrition counseling in pregnancy and (2) counseling, including checking on the status of exclusive breastfeeding and complementary feeding. Checklists to review quality of counseling were introduced but implemented in pilot geographies only.

All countries use District Health Information System2 (DHIS2). However, in none of the countries is the system computerized end-to-end. Data are reported from the lowest tier of health facilities and consolidated at sub-national levels before reporting at the National level. In all countries, reporting by community health workers associated with the lowest tier health facility is done manually. DHIS2 is a health information system; hence, modules on nutrition themes need to be created for the software. Recently, in collaboration with UNICEF, DHIS2 released the NUT digital data pack, which bridges the gap in availability of nutrition-focused modules.

To generate data on quality of counseling for local decision making, mApps for use by community health workers and their supervisors have been successfully tested in Bangladesh and India. As data on MIYCN counseling has limited availability, not much can be said on its usage to strengthen MIYCN programming at country level.

### Financing for MIYCN counseling

It was challenging to get information on costing or status of funding for MIYCN counseling in South Asian countries. Four papers were sourced through key informants covering Bangladesh, India, Nepal, and Sri Lanka. The spread of MIYCN counseling services across several programs made it challenging to infer status of MIYCN counseling financing. An India study provided cost estimates for scaled-up MIYCN counseling services compared with food supplementation, health services, micronutrient supplementation, deworming and maternity benefit schemes. Attempts to review budgets and expenditure for trainings which include MIYCN counseling did not yield usable information.

## Discussion

This analysis provides insights into specific areas to strengthen MIYCN counseling related to (1) policy, (2) workforce, (3) counseling content and materials, and (4) information systems. The analysis on coverage, continuity, intensity, and quality of MIYCN counseling for these seven countries provides the status of MIYCN counseling and facilitates interpretation of findings on the four system inputs listed above.

The analysis highlights gaps in adapting global recommendations on counseling on seven themes. These seven themes are: (1) diet guidance by gestational age (unavailable in Bhutan, Nepal, Pakistan, Sri Lanka), (2) pre-pregnancy BMI-based gestational weight gain counseling (unavailable in all countries except Maldives), (3) specificity of type and duration of physical activity in pregnancy and during breastfeeding (unavailable in all countries except Maldives), (4) IFA dosage, consumption and managing side-effects in breastfeeding mothers and children (child IFA supplementation counseling relevant for Bhutan, India, Maldives, Nepal and Pakistan; unavailable in all except India), (5) counseling undernourished pregnant women and breastfeeding mothers (screening for thinness unavailable in Bhutan, India), (6) community-based management of uncomplicated SAM and MAM and (7) management of overweight and obesity among all target groups (unavailable in all countries except Maldives and Sri Lanka) (18–26).

At least four ANC contacts with skilled or unskilled providers can be made more impactful to improve maternal nutrition counseling. Having the first ANC contact in the first trimester of pregnancy can improve the earlier reach of counseling, particularly in Bangladesh, Bhutan, and Pakistan. At least four ANC contacts have no correlation with early initiation of breastfeeding in four of the seven countries: Bangladesh, Nepal, Pakistan, and Sri Lanka (OR 1.0, 1.2, 1.0, 1.0, respectively). Counseling and support for breastfeeding immediately after birth is deficient across South Asia except in Sri Lanka. Despite increasing institutional delivery rates, the proportion of mothers initiating breastfeeding within an hour of birth is lower across all countries, except Bangladesh (29–36). Some reasons for delayed initiation of breastfeeding in institutional deliveries especially in the private sector could be the lack of implementation support for early initiation of breastfeeding in health facilities, including rooming-in, limited training of health staff on criticality of early initiation of breastfeeding and how to support mothers to breastfeed, and increased c-section rates coupled with a lack of guidance and systems to initiate breastfeeding early after c-section (41, 42). South Asian countries need to review counseling content related to breastfeeding for pregnant women, reaching more family influencers and strengthening delivery care systems to support early initiation of breastfeeding for both home and institutional deliveries. GMP services need to be strengthened with referral and follow-up for children with MAM and SAM and have a dedicated management package for children affected by overweight or obesity. In Bangladesh, layering GMP services on the established EPI platform is being attempted as recommended in recent health and nutrition program review (43).

The data deficiency on assessing coverage, continuity, intensity and quality for complementary feeding behaviors is a serious concern in five of seven countries, with the exception of India and Nepal. In India and Nepal, health worker contacts did not correlate with timely initiation of complementary feeding (India OR 1.0, Nepal OR 1.7). This could partly be explained by the quality of data available for these contacts. In India, health worker contact data were not specifically for nutrition counseling, while in Nepal the period of recall for health worker contact was one year opposed to three months for India. In India, health worker contacts did correlate with children being fed a more diverse diet, but the strength of the correlation was the same as that for other influencers such as being an urban resident or belonging to a household in the richest income quintile.

MIYCN counseling is mostly an add-on role for community health workers and assumed role for facility staff who contact pregnant women, breastfeeding mothers, and infants and young children. The review highlights the varying qualification criteria and pre-service trainings for community health workers across the seven countries. Except in Bangladesh and India, most country nutrition training packages for health workers cover IYCF only, and within that, mainly focus on breastfeeding. Countries need training curriculum on maternal nutrition and complementary feeding to cover the full spectrum of counseling under MIYCN. This applies to both community-based and facility-based service providers. As noted in the review, graduate training of nurses and doctors also lack focus on MIYCN counseling, particularly on maternal nutrition. Community health workers face similar socio-cultural-gender barriers as pregnant women and mothers. Training should cover overcoming these barriers and negotiating behavior change with male members of the family.

Delays in roll-out of pre-service or entry-level trainings, resource constraints in organizing any refresher trainings, and poor quality of trainings are systemic problems in the region which also affect training on MIYCN counseling. Supervisors can play a critical role to hone service providers’ MIYCN counseling skills as there is opportunity to directly observe challenges faced by service providers and provide hands-on, continuing support. Ideally, country training plans should cover joint trainings of service providers and supervisors on MIYCN counseling with supervisors trained on how to provide feedback and support for different types of challenges: technical, community resistance, supply issues, and others. If resource limitations impede the organization of joint trainings, trainings for supervisors should be prioritized. Technology-enabled tools to ease supportive supervision are available for adaptation (44). The Sahyog mobile application for supervisors under ICDS is being used in one of the largest states in India, Uttar Pradesh, and being scaled up in other states. The application enables efficient scheduling of supervision visits; guided observations and observation recording for follow-up with supervisees; learning aids for supervisors and supervisees; and the generating performance data instantly for decision making.

Across countries there is a disproportionate availability of materials with standard messages on breastfeeding and eating foods from diverse food groups. Most of these materials are in print format and suited to rural audiences. Materials need to be contextualized especially for a non-rural audience. While urban poor mainly comprise migrants from rural areas or smaller towns and can relate to content, access to food resources and health care service providers is starkly different for urban poor than rural residents. The urban middle- and higher-income population is influenced by social media, which may provide incorrect information and include aggressive marketing of food products including for infants. Influence of social media on food preferences was not part of the scope of this study although it is an emerging area of concern. Food items included in counseling materials for complementary feeding should consider costing, problems in sourcing raw ingredients, and cooking time for mother and family. Cooking practices in some countries lean toward use of high fat, sugar, and salt ingredients, which should be checked for in recipes suggested to women and children. There is opportunity to enrich growth charts with standard messages for mother/caregiver based on child’s nutritional status (green, yellow or red zone). Beyond use of counseling materials and demonstrations, discussions around important life events like pregnancy announcements or the initiation of complementary feeding have been successful in changing MIYCN behaviors in South Asia due to wider community engagement, the engagement of males, and less dependance on literacy (45, 46).

In the South Asian context, cultural and gender norms in most countries limit access to information for women who are the primary clients for services in the first 1,000 days (47). These norms adversely affect translation of information into practice even when available. However, except for audio-visual materials from Bangladesh and Nepal, messaging was targeted mostly at women. Nuanced material is needed to cover all recommended counseling and reach the full range of target groups, which include families (husband, fathers, parents-in-law) of direct clients as well as community leaders. These family and community influencers shape health and nutrition behaviors, including types of foods consumed and uptake of nutrition services by pregnant women and children. Most countries have done well in developing materials in local languages.

In all countries, MIYCN counseling materials need to be organized by platforms and most appropriate service provider to identify need for any new materials. Service providers have varying qualifications and capacities which need to be considered when developing new materials. How users will be trained on use of new materials should be planned in line with ongoing capacity building efforts on MIYCN. In Bangladesh and India, community volunteers such as self-help group members engaged in MIYCN counseling should be considered when organizing materials by service providers. With the increasing reach of internet service and hardware, web-based counseling through artificial intelligence is gaining momentum. There is opportunity to transform available print materials to electronic or creating new materials for tech-based dissemination.

MIYCN counseling is a service that requires investments in time, capacity building, counseling materials, and organization of events, all of which should be defined across community- and facility-based platforms. Targets for counseling contacts and events especially for community-based platforms are needed in country plans and budgets. Each country has established platforms for delivering counseling services but miss monitoring delivery of counseling services. A curated set of indicators that provide the most relevant data to strengthen counseling services at local level are needed. These indicators should clearly establish the reach of counseling services platforms, especially for infants and young children and as well as an assessment of quality of counseling. Notable counseling related indicators being used in few South Asian countries are on consumption of IFA and increasing dietary diversity in pregnancy, breastfeeding and GMP. Monitoring should include verification of information by direct observations of behaviors by service providers such as observing breastfeeding or a child feeding session. A few of these indicators may be used for upward reporting.

### Limitations

The analysis on coverage, continuity, intensity, and quality of MIYCN counseling is based on proxy indicators of maternal and child contact points due to the unavailability of direct MIYCN counseling indicators. Most countries are working to integrate nutrition services with these contact points. In DHS, the duration of recall for most of these contacts varied from three to five years across countries. To limit bias due to variable recall and have comparable data, the duration of recall was set at three years during analysis. The definition of health worker contacts for infants and children varied for India and Nepal and this data is not comparable. KIIs covered representatives from national government, training institutes, and development partners to gain insights on policy, planning and implementing MIYCN counseling programs at a high level. Service provider and client perspectives on MIYCN counseling were not in scope of this study but are critical for a comprehensive assessment of MIYCN counseling services; country-level assessments should include them as key respondents. In Balochistan, Pakistan, KIIs could not be completed as the areas was inundated with floods during the time of data collection. Counseling materials were sourced from websites and key informants. These do not cover the whole range of materials available, especially in larger countries (Bangladesh, India, and Pakistan). Awareness generation materials meant exclusively for dissemination through mass media and social networks were not included in this study.

### Recommendations

Based on these findings, countries in South Asia need to review guidelines on MIYCN counseling to align with global recommendations. Country policy and planning should recognize MIYCN counseling as a service with defined set of activities, across different platforms. MIYCN counseling roles need to be rationalized against job responsibilities, qualifications, and training of service providers. Training curriculum needs to be updated for all cadres required to provide MIYCN counseling as per global recommendations. Health worker trainings on MIYCN counseling need to be backed with supportive supervision by skilled supervisors. Counseling materials need to be mapped for coverage of content and target groups, types of users, and format to determine need for any additional materials. Use of counseling materials needs to be restructured to deliver the right message, by the right provider, and at the right time. Countries need to establish a system to monitor MIYCN counseling services and set accountability mechanisms to deliver counseling as is done to deliver nutrition commodities.

## Data availability statement

Publicly available datasets were analyzed in this study. This data can be found here: https://dhsprogram.com/data/available-datasets.cfm.

## Author contributions

AB, VS, ZM, SG, and TF were involved in conceptualization and methodology. AB and SG supervised data collection. AB and KS were involved in data analysis. AB, VS, and TF led the drafting of the manuscript. VS, ZM, and TF were involved in funding acquisition and project administration. All authors provided technical input, read, and agreed to the published version of the manuscript.

## Conflict of interest

The authors declare that the research was conducted in the absence of any commercial or financial relationships that could be construed as a potential conflict of interest.

## Publisher’s note

All claims expressed in this article are solely those of the authors and do not necessarily represent those of their affiliated organizations, or those of the publisher, the editors and the reviewers. Any product that may be evaluated in this article, or claim that may be made by its manufacturer, is not guaranteed or endorsed by the publisher.
