# Supplementary material for: Right message, right medium, right time: powering counseling to improve maternal, infant, and young child nutrition in South Asia
Source: Front Nutr. 2023 Sep 4;10:1205620. doi: 10.3389/fnut.2023.1205620 (PMC10512175; doi:10.3389/fnut.2023.1205620)
Supplement: Supplementary file 1 [file Data_Sheet_1.docx]

**Supplementary table 1. Odds of early initiation of breastfeeding by background characteristics, receipt of at least four ANC contacts, institutional delivery and women’s access to mid-mass media**

|  | **BGH** |  | **BHN** |  | **IND** |  | **MAL** |  | **NPL** |  | **PAK** |  | **SL** |  |
| --- | --- | --- | --- | --- | --- | --- | --- | --- | --- | --- | --- | --- | --- | --- |
| N | 5012 | 95% CI | 887 | 95% CI | 122458 | 95% CI | 1693 | 95% CI | 2761 | 95% CI | 6276 | 95% CI | 4635 | 95% CI |
| Rural vs Urban | 1.0 | 0.8-1.2 | 0.9 | 0.3-2.3 | 1.0 | 1.0-1.1 | NA |  | 1.0 | 0.8-1.3 | 1.1 | 0.9-1.4 | 0.8 | 0.5-1.2 |
| Age <20 yrs vs >=20 yrs | 1.2* | 1.0-1.5 | 6.0*** | 1.8-20.0 | 1.0 | 0.9-1.0 | 2.7 | 0.7-10.1 | 1.0 | 0.8-1.4 | 0.9 | 0.6-1.2 | 0.7 | 0.2-2.4 |
| Wealth quintile poorest vs |  |  |  |  |  |  |  |  |  |  |  |  |  |  |
| poorer | 1.1 | 0.8-1.4 | 1.1 | 0.5-2.2 | 0.9*** | 0.8-0.9 | 1.5 | 0.8-2.8 | 0.9 | 0.6-1.2 | 0.8 | 0.6-1.1 | 1.5 | 0.9-2.7 |
| middle | 1.1 | 0.8-1.4 | 2.1 | 0.7-6.4 | 0.8*** | 0.8-0.9 | 1.3 | 0.7-2.3 | 0.8 | 0.5-1.1 | 0.9 | 0.6-1.2 | 1.2 | 0.7-2.1 |
| richer | 0.9 | 0.7-1.2 | 1.4 | 0.4-4.1 | 0.9*** | 0.8-0.9 | 1.2 | 0.7-2.2 | 0.9 | 0.6-1.2 | 0.9 | 0.6-1.2 | 1.2 | 0.7-2.0 |
| richest | 1.2 | 0.9-1.6 | 1.2 | 0.3-4.4 | 1.0 | 0.9-1.0 | 1.3 | 0.7-2.4 | 0.6** | 0.4-1.0 | 1.1 | 0.7-1.7 | 1.2 | 0.7-2.1 |
|  |  |  |  |  |  |  |  |  |  |  |  |  |  |  |
| At least 4ANC = yes | 1.2 | 1.0-1.4 | 4.9*** | 2.6-9.2 | 1.4*** | 1.3-1.4 | 1.6** | 1.0-2.6 | 1.1 | 0.8-1.4 | 1.0 | 0.8-1.3 | 1.2 | 0.1-9.5 |
| Public vs private sector provider | 1.0 | 0.8-1.2 | NA |  | 0.9*** | 0.8-0.9 | 0.9 | 0.5-1.5 | 0.9 | 0.6-1.2 | 0.7*** | 0.5-0.9 | 1.0 | 0.7-1.5 |
| Skilled vs unskilled health care provider | 1.2 | 0.7-1.5 | NA |  | 1.0 | 0.9-1.0 | NA | NA | 0.7 | 0.4-4.3 | 1.3 | 0.6-2.5 | 0.9 | 0.3-2.5 |
| Institutional delivery public vs |  |  |  |  |  |  |  |  |  |  |  |  |  |  |
| private | 0.6*** | 0.5-0.8 | NA |  | 0.7*** | 0.6-0.7 | 1.0 | 0.6-1.6 | 0.7*** | 0.2-1.8 | 0.9 | 0.7-1.2 | 1.0 | 0.5-2.0 |
| home | 1.6*** | 1.2-2.0 | NA |  | 0.8*** | 0.7-0.8 |  |  | 0.5*** | 0.4-0.7 | 1.1 | 0.8-1.5 |  |  |
| Own mobile phone = yes | 1.0 | 0.9-1.2 | NA |  | 0.9** | 0.8-1.0 | 0.7 | 0.3-1.9 | 0.9 | 0.7-1.2 | 1.0 | 0.7-1.2 | 0.9 | 0.6-1.5 |
| Read newspaper at least once a week = yes | 0.9 | 0.6-1.4 | NA |  | 1.0** | 1.0-1.2 | 1.5** | 1.0-2.2 | 0.9 | 0.5-1.6 | 1.1 | 0.7-1.7 | 1.2 | 0.9-1.7 |
| Listen to radio at least once a week = yes | 0.9 | 0.5-1.5 | NA |  | 1.0 | 0.9-1.1 | 1.0 | 0.6-1.4 | 0.9 | 0.7-1.2 | 1.2 | 0.8-1.9 | 1.0 | 0.7-1.4 |
| Watch television at least once a week = yes | 0.9 | 0.7-1.1 | NA |  | 1.3*** | 1.2-1.3 | 0.8 | 0.5-1.4 | 1.0 | 0.8-1.3 | 1.0 | 0.8-1.3 | 0.9 | 0.6-1.4 |
| Total number of influencers | 3 |  | 2 |  | 10 |  | 2 |  | 3 |  | 1 |  | 0 |  |

* p<0.1 **p<0.05 *** p<0.01 NA: Not available ^#^

**Supplementary table 2. Timing of PNC contact**

|  | **BGH** | **BHN** | **IND** | **MAL** | **NPL** | **PAK** | **SL** |
| --- | --- | --- | --- | --- | --- | --- | --- |
| **<4 hours** | NA | NA | 73.2 | 65.6 | NA | 52.9 | 91.5 |
| **4-23 hours** | NA | NA | 3.5 | 9.8 | NA | 6.4 | 5.9 |
| **1-2 days** | NA | NA | 4.8 | 4.8 | NA | 1.9 | 1.9 |
| **Withing 2 days** | 52.1 | 74 | 81.5 | 80.2 | 55.1 | 61.2 | 99.3 |

NA: Not available

**Supplementary table 3. Gender review of counselling materials**

| Materials for pregnant women | **BGH** | **BHN** | **IND** | **MAL** | **NPL** | **PAK** | **SRL** |
| --- | --- | --- | --- | --- | --- | --- | --- |
| **Total number of materials included for gender review*** | **12** | **8** | **35** | **3** | **4** | **3** | **1** |
| Equal number of men and women depicted | 10 | 0 | 35 | 2 | 4 | 0 | 1 |
| Equal number of boys and girls depicted | NA | NA | 32 | 3 | 4 | 3 | NA |
| Any images of adults/children with disability | 0 | 0 | 0 | 0 | 0 | 0 | 0 |
| Images reflect national diversity | NA | NA | 0 | NA | NA | NA | NA |
| Images don’t enforce stereotypes (e.g. girls/ women doing household chores while boys playing, using gadgets) | 12 | 6 | 35 | 0 | 4 | 0 | 1 |
| Professionals – doctors, nurses, counsellors – are either women or both men and women depicted equally | 6 of 6 | 5 Of 5 | 31 of 31 | NA | 4 | 1 of 2 | NA |
| Leaders- village head/ elected officials are either women or both men and women depicted equally | NA | NA | 7 of 7 | NA | NA | NA | NA |
| Use of gender-neutral pronouns or he/she | 12 | 2** | 35 | 3 | 4 | 3 | 1 |
|  |  |  |  |  |  |  |  |
| Materials for breastfeeding mothers | **BGH** | **BHN** | **IND** | **MAL** | **NPL** | **PAK** | **SRL** |
| **Total number of materials included for gender review*** | **8** | **8** | **29** | **3** | **4** | **6** | **1** |
| Equal number of men and women depicted | 8 | 0 | 29 | 2 | 4 | 1 | 1 |
| Equal number of boys and girls depicted | NA | NA | 25 | 3 | 4 | 4 | 1 |
| Any images of adults/children with disability | 0 | 0 | 0 | 0 | 0 | 0 | 0 |
| Images reflect national diversity | NA | NA | 0 | NA | NA | 6 | NA |
| Images don’t enforce stereotypes (e.g. girls/ women doing household chores while boys playing, using gadgets) | 0 | 6 | 29 | 3 | 4 | 0 | 1 |
| Professionals- doctors, nurses, counsellors are either women or both men and women depicted equally | 4 of 5 | 5 Of 5 | 25 of 25 | NA | 4 | 1 of 2 | NA |
| Leaders- village head/ elected officials are either women or both men and women depicted equally | NA | NA | 7 of 7 | NA | NA | NA | NA |
| Use of gender-neutral pronouns or he/she | NA | 2** | 29 | 3 | 4 | 6 | 1 |
|  |  |  |  |  |  |  |  |
| Materials for mothers/caregivers of children 6-23 months | **BGH** | **BHN** | **IND** | **MAL** | **NPL** | **PAK** | **SRL** |
| **Total number of materials included for gender review*** | **11** | **14** | **24** | **4** | **6** | **5** | **4** |
| Equal number of men and women depicted | 8 of 8 | 2 | 24 | 2 | 6 | 1 | 4 |
| Equal number of boys and girls depicted | 8 | 2 | 21 | 4 | 6 | 2 | NA |
| Any images of adults/children with disability | 0 | 0 | 0 | 0 | 0 | 0 | 0 |
| Images reflect national diversity | NA | NA | 0 | NA | NA | 5 | 0 |
| Images don’t enforce stereotypes (e.g. girls/ women doing household chores while boys playing, using gadgets) | 6 | 14 | 24 | 4 | 6 | 0 | 4 |
| Professionals- doctors, nurses, counsellors are either women or both men and women depicted equally | 4 of 5 | 2 of 2 | 20 of 20 | 0 of 1 | 6 | NA | 0 of 1 |
| Leaders- village head/ elected officials are either women or both men and women depicted equally | NA | NA | 7 of 7 | NA | NA | NA | NA |
| Use of gender neutral pronouns or he/she | 11 | 14 | 24 | 4 | 6 | 5 | 4 |

*Numbers may be lower than the total reviewed for thematic coverage as materials with only images of foods were excluded due to no relevance in analysis ** Due to local dialect and/or no audio, evaluator could only assess 2 videos NA: Not applicable
